# Relationship between Areas of Cognitive Functioning on the Mini-Mental State Examination and Crash Risk

**DOI:** 10.3390/geriatrics3010010

**Published:** 2018-03-06

**Authors:** Carrie Huisingh, Virginia G. Wadley, Gerald McGwin, Cynthia Owsley

**Affiliations:** 1 Department of Ophthalmology, School of Medicine, University of Alabama at Birmingham, 700 S. 18th St., Suite 609, Birmingham AL 35294, USA; owlsey@uab.edu; 2 Division of Gerontology, Geriatrics, and Palliative Care, School of Medicine, University of Alabama at Birmingham, Birmingham AL 35294, USA; vwadley@uab.edu; 3 Department of Epidemiology, School of Public Health, University of Alabama at Birmingham, Birmingham AL 35294, USA; mcgwin@uab.edu

**Keywords:** MMSE, cognitive domains, older drivers, crash, orientation to place, spatial orientation

## Abstract

Previous studies have suggested that the pattern of cognitive impairment in crash-involved older drivers is different from non-crash-involved older drivers. This study assessed the relationship between seven areas of cognitive functioning (orientation to time, orientation to place, registration, attention and calculation, recall, language, and visual construction) on the Mini-Mental State Examination (MMSE) collected at baseline and rates of future crash involvement in a prospective population-based sample of older drivers. Motor vehicle collision (MVC) involvement was obtained from the Alabama Department of Public Safety. Poisson regression was used to calculate crude and adjusted rate ratios (RR). Older drivers having difficulties in place orientation were more than 6 times (95% CI 1.90–19.86) more likely to be involved in a future crash (adjusted RR = 6.14, 95% confidence interval (CI) 1.90–19.86) and at-fault crash (adjusted RR = 6.39, 95% CI 1.51–27.10). Impairment in the other cognitive areas was not associated with higher rates of crash or at-fault crash involvement. The findings were validated in an independent sample of high-risk older drivers and a similar pattern of results was observed. Spatial orientation impairment can help identify older drivers who are more likely to crash in the future.

## 1. Introduction

There are currently about 32 million adults aged 70 years and older in the United States with over 80% of them licensed to drive [1,2]. In 2015, there were 6165 older adults killed and an estimated 240,000 injured in motor vehicle traffic crashes in the United States [3]. As more of the U.S. population reaches age 70 and older, it becomes increasingly important to identify risk factors that elevate crash risk for older drivers because the risk of crashing increases rapidly after age 70 per mile driven [4]. One contributor to the high rates of crash involvement is cognitive dysfunction. Older drivers with cognitive impairment due to dementia are more than twice as likely to be crash-involved compared to those without dementia [5,6,7]. Depending on the severity, dementing diseases might be suspected of increasing the risk of crashes because they affect memory, language, visuo-spatial processing, and other higher-order cognitive functions such as judgment and perception that are used to safely drive and control a vehicle [5,6,7,8].

The Mini-Mental State Examination (MMSE) is a widely used tool to assess general cognitive function in both routine clinical practice and research settings [9]. The MMSE was designed to assess different aspects of function known to be impaired in dementia including orientation to time, orientation to place, word registration, attention and concentration, recall, language, and visual construction. The total MMSE score correlates well with dementia progression [10,11], so it would seem logical that MMSE impairment would also correlate well with crash risk. However, the association between MMSE impairment status and crash risk has been mixed with most studies showing a null association [5,8,12,13,14,15,16,17,18,19,20,21,22,23,24], others a positive association [25,26], and one with a protective association [27]. These results were based on the overall MMSE score, not the specific cognitive aspects of mental functions the MMSE assesses, so the inconsistency may be due to variation in the cognitive symptom profile from sample to sample. That is, there may be a differential relationship between the specific MMSE cognitive domains and crash risk. In a meta-analysis by Reger et al. [28] that examined the relationship between neuropsychological tests and driving ability of persons with dementia, the researchers concluded that when taken as a whole, the neuropsychological test battery was not strongly correlated to driving performance. However, when tests measuring specific areas of function were separated from the entire test battery, the correlation between driving performance and cognitive testing improved [28].

Examining the individual cognitive domains of the MMSE may provide useful information on the type of cognitive functions associated with crash risk [29]. Poor performance on the visual construction cognitive domain has been positively associated with driving cessation [24,30], poor driving performance [31], and crash involvement [26,32]. Impairment on the attention and concentration domain has been associated with prior crash involvement [25] in cross-sectional analyses. In prospective studies, none of the MMSE cognitive domains were associated with an increased risk of crash involvement [13,23]; however, these studies defined crash outcomes based on self-report which may be subject to recall bias. Therefore, the objective of the current study was to assess the relationship between specific areas of function on the MMSE and future rates of crash involvement in a population-based sample of older drivers.

## 2. Materials and Methods

### 2.1. Study Participants

#### 2.1.1. Licensure Sample

The Licensure sample was a population-based sample of older drivers ages >70 years old who resided in north central Alabama [33]. Potential participants were randomly identified from contact information available through a list of persons in this geographic area obtained from a direct marketing company (Pinpoint Technologies, Tustin, CA, USA). The driver’s license status was confirmed through the Alabama Department of Public Safety and those who did not hold Alabama licenses were eliminated. Potential participants were randomly selected from the final list and contacted by letter that was followed-up by a phone call. Those who stated they had an Alabama license, had driven within the last three months, and spoke English were eligible to participate. Participants (*N* = 2000) were enrolled from October 2008 through August 2011. Participants completed a single in-person visit at the Clinical Research Unit at the University of Alabama at Birmingham and were followed-up with telephone calls at one-year intervals for three subsequent years.

#### 2.1.2. KEYS Sample

KEYS (Knowledge Enhances Your Safety) was a randomized, controlled, single-masked intervention evaluating an educational program that promoted strategies to enhance driver safety compared to usual care (a comprehensive eye examination) among 403 high-risk older drivers, as described in detail elsewhere [34]. Briefly, licensed drivers aged 60 years and older were eligible if they had been the driver in a crash in the prior year, drove at least 5 days per week or at least 100 m per week or both, had a Mini-Mental State Examination (MMSE) score of 23 or greater, and had visual impairment. The reason for the MMSE cut-off was because KEYS was an educational intervention, and those with significant cognitive impairment may have failed to benefit from the educational intervention. Visual impairment was defined as (1) visual acuity (habitual, binocular) between 20/30 and 20/60 as measured by the Early Treatment of Diabetic Retinopathy (ETDRS) chart [35], or (2) an impairment in the useful field of view test [36] or both. Eligible and consenting participants were randomized to usual care versus usual care plus the educational intervention. Participants were enrolled from March 1998 through February 2000. Similar to Licensure, participants completed the initial study visit in the Clinical Research Unit at the University of Alabama at Birmingham Department of Ophthalmology and then completed telephone interviews at 6-month intervals for 2 years.

### 2.2. Measurements

At the baseline visit, standard protocols for the MMSE were administered in both studies using the alternative administration of the attention and concentration domain, which was assessed based on spelling “world” backward only, rather than serial seven calculations. The MMSE scale ranges from 0 to 30; the higher the score, the better the cognitive performance. For both studies, impaired cognitive status (overall) was defined as a MMSE score of <24 [9]. We then grouped the questions into seven cognitive functions. Scores within each function were summed, giving one point to each correct response, and dichotomized as follows: Orientation to time (0–3 vs. 4–5 points), orientation to place (0–3 vs. 4–5 points), registration (immediate recall) (0–2 vs. 3 points), attention and calculation (0–3 vs. 4–5 points), recall (0–2 vs. 3 points), language (0–4 vs. 5–8 points), and visual construction (0 vs. 1 point).

Information on demographic characteristics (age, gender, race, education) and visual acuity was assessed in both studies. Visual acuity was assessed using the Electronic Visual Acuity (EVA) [37] system in Licensure and using the Early Treatment of Diabetic Retinopathy (ETDRS) chart [35] in KEYS. The EVA has been validated against the ETDRS chart [37]. In both studies, visual acuity testing was administered under binocular viewing done under habitual correction (i.e., the correction the participant used when driving). Visual acuity was expressed as logarithm of the minimum angle resolvable (logMAR) and impairment was defined as 0.3 logMAR or greater.

The primary outcome for the present analysis was number of motor vehicle collisions during follow-up, which was measured in both studies. Information on collisions in which the participant was the driver was obtained from accident reports provided by the Alabama Department of Public Safety. Of relevance, the date of the collision and at-fault status according to the police officer at the scene was indicated on the report.

An estimate of driving status and driving exposure information during the past year was collected in both studies using the Driving Habits Questionnaire, a valid and reliable instrument [38]. In Licensure, data were collected at baseline and during the annual telephone interviews. In KEYS, information was collected at the initial visit and then by telephone at 6-month intervals for the 2 years subsequent to randomization. This measure was used to calculate annual miles driven and identify participants who were current drivers and therefore were considered at risk. In Licensure, five participants were excluded from the analysis because they reported being a current driver at baseline, but during the follow-up reported a date of driving cessation prior to study entry. Therefore, the current analysis included data from 1995 Licensure participants. The mileage reported during each interview was summed while the participants were considered at-risk to calculate total mileage during the study. Participants were considered no longer at risk due to driving cessation or death.

Date of death was confirmed by searching the Social Security Death Index or newspaper obituaries. Participants were considered at-risk for MVC involvement until the earliest of death, driving cessation, or the end of each study period.

The Institutional Review Board at the University of Alabama at Birmingham approved this study.

### 2.3. Statistical Analysis

Demographic, visual, driving, and MMSE domain characteristics were compared between Licensure and KEYS samples. T-tests and chi-square tests were used to compare continuous and categorical variables, respectively. Poisson regression was used to estimate the association between each area of cognitive functioning on the MMSE and future crash rates among Licensure participants. Separate models were used to examine the association with future at-fault crash rates. An offset term was used to account for annual miles driven. Models were adjusted for age, gender, race, education, and visual acuity impairment status. These analyses were then replicated using the follow-up data from KEYS. Models were adjusted for the same covariates as in Licensure. In KEYS, models were not adjusted for randomization assignment since this was not related to any of the independent variables, so was not considered a confounder. The adjusted rate ratio (RR) and 95% confidence interval (CI) are reported for the MMSE overall and for each area.

## 3. Results

### 3.1. Baseline Characteristics in Licensure

Table 1 presents study-specific demographic and visual characteristics. On average, participants were aged 77.2 years (SD 5.0), ranging from 70 to 92. Participants were 43.7% female and 81.9% White. Approximately two-thirds of the sample had a high school education (or General Educational Development (GED) equivalent) or above. Eight percent had impaired visual acuity. Overall, 2.3% of the sample had an MMSE score <24 and the prevalence of impairment in each area of cognitive function varied. Just over half the sample (50.7%) were impaired on delayed recall, 11.2% in visual construction, and 4.8% in attention and concentration. Less than 2% were impaired on orientation to time (1.4%), orientation to place (0.2%), registration (0.9%), and language (0.3%).

### 3.2. Association between Area of Cognitive Function and Rates of MVC in Licensure

During the follow-up period, 249 (12.5%) drivers were involved in any crash and 129 (6.5%) were involved in an at-fault crash. After adjusting for age, gender, race, education, and visual acuity impairment status, impairment on the MMSE overall was not significantly associated with the crash rate (RR = 1.43, 95% CI 0.76–2.66) (Table 2). When the MMSE was broken down into the seven areas of cognitive function, the rates of crash involvement were 6.14 (95% CI 1.90–19.86) times higher for those with impairment in orientation to place compared to those without impairment. The associations between impairment in the other six functions and rate of crash involvement were not statistically significant (Table 2). A similar pattern of results was found with at-fault crashes. Impairment on the MMSE overall was not significantly associated with the at-fault crash rate (RR = 1.83, 95% CI 0.87–3.88). For those with impairment in orientation to place, rates of at-fault crash involvement were 6.39 (95% CI 1.51–27.10) times higher compared to those without impairment. The associations between impairment on the other functions and rate of at-fault crash involvement were not statistically significant. Zero at-fault crashes occurred among those with impairment on the language function, so rates could not be calculated.

### 3.3. Validation of Findings from Licensure in the KEYS Dataset

The KEYS sample differed from the Licensure sample (Table 1). The mean participant age was younger (73.5 vs. 77.2 years) and a lower percent were female (31.0% vs. 43.7%) and White (77.1% vs. 81.9%) compared to the Licensure sample. A higher percentage of participants had a high school education or above (78.1% vs. 68.4%) and KEYS participants reported driving more miles per year than Licensure participants. Visual acuity impairment was similar in both samples. Despite the eligibility criterion of having a MMSE score of 23 or greater in KEYS, the sample had 3.8% with an MMSE score of <24 compared to 2.3% in the Licensure sample. In general, the pattern of impairment on the specific areas of cognitive function was similar in the KEYS sample. About two-thirds of the sample (64.2%) were impaired on the delayed recall domain. Visual construction was impaired in 15.9%. About 10% were impaired on the registration and attention and concentration domains. About 1% were impaired on orientation to time (1.3%) and orientation to place (1.0%) domains. No participants were impaired on the language domain.

Among the KEYS participants, 74 (18.6%) were crash-involved and 48 (12.1%) were deemed to be at-fault for the crash during the follow-up period. After adjusting for age, gender, race, education, and visual acuity impairment status, impairment on the MMSE overall was not significantly associated with the crash rate (RR = 0.63, 95% CI 0.15–2.60) (Table 2). When the MMSE was broken down into the seven areas of cognitive functioning, the rates of crash involvement were 6.37 (95% CI 2.26–17.92) times higher for those with impairment in the orientation to place compared to those without impairment. The associations between impairment on the other six areas of cognitive function and rate of crash involvement were not statistically significant (Table 2). A similar pattern of results was found with at-fault crashes. Impairment on the MMSE overall was not significantly associated with the at-fault crash rate (RR = 1.05, 95% CI 0.25–4.44). Impairment on the orientation to place function was significantly associated with increased rates of at-fault crash involvement (RR = 10.58, 95% CI 3.67–30.54) compared to those without impairment. The associations between impairment on the other areas of function and rate of at-fault crash involvement were not statistically significant. Rates could not be calculated for those with and without impairment in the language function. 

Finally, as an aid to interpreting our findings, we examined the mean total MMSE score achieved by those with and without impairment in the seven areas of cognitive functioning (Table 3). Those with impairment in the spatial orientation and language domains had far worse total MMSE scores than those with impairments in the remaining domains.

## 4. Discussion

The purpose of this study was to examine the association between the cognitive domains on the MMSE and rates of crash and at-fault crash involvement. When the cognitive aspects of mental function were parsed out, rates of future crash and at-fault crash involvement were more than six times higher for those with impaired spatial orientation compared to those without impairment. The other areas of cognitive function were not associated with crash or at-fault crash involvement after adjusting for several potentially confounding variables. The findings were then validated in an independent sample of high-risk older drivers and a similar pattern was observed, suggesting the findings are robust.

Our results suggest that impairment in the orientation to place domain is particularly germane to understanding crash risk in older drivers. The precise mechanism that underlies this association is not clear. One possibility is that impaired spatial orientation may be a marker of dementia severity. Supporting this interpretation, the mean MMSE score of participants with impaired spatial orientation was quite low at 18 points out of 30 (Table 3). Disorientation is a major component of dementia including Alzheimer’s disease, and is an indicator of decline in the disease spectrum [29,39,40]. In fact, the evolution of cognitive decline over time in those with Alzheimer’s disease starts with decline in word recall and orientation to time followed by attention and concentration, orientation to place, language, visual construction, and finally registration [29]. If impaired spatial orientation is a marker of advanced disease severity, it would be reasonable to expect functions that become impaired at a more severe stage of disease to also be associated with crash risk. However, in the current study, none of the other domains were associated with MVC involvement. More than two decades ago, studies on driving safety by Marottoli et al. and Johansson et al. reported an association with the copy design task (draw intersecting polygons) [26,32], but this association has not been observed in any subsequent reports [13,23,24,25]. While this task requires higher-order thinking skills necessary for safe driving, it may be that older drivers with some visuospatial or motor difficulty were already making adjustments in their driving behavior by limiting their driving to less challenging conditions, resulting in no association with crash events. The fact that impaired registration, one of the last functions to decline in dementia, appeared to have a protective association for crash risk, albeit not statistically significantly, supports this possibility.

Another possibility is that loss of sense of place may represent a confused state which in turn may impede understanding of road sign or controlled intersection instructions and may contribute to the incidence of crash involvement [41]. Impairment in spatial orientation is associated with other types of health outcomes in older adults such as poor rehabilitation outcomes [42], increased falls risk [41,43], as well as the development of delirium [41]. It is interesting to note that falls have also been associated with increased MVC risk [32,44,45], so there may be shared risk factors for mobility impairment. 

From a clinical perspective, examining the specific areas of function on the MMSE may be important in the development of brief tests for primary care clinicians and geriatricians [25]. Physicians are often asked to assess an older adult’s fitness to drive, but do not always know what cut-point to use to initiate that conversation. Results from the current study suggest that noting the areas in which patients “lose” their points on the MMSE may assist with identifying high-risk older drivers. For those responding incorrectly to two or more questions on the spatial orientation domain, the clinician should consider discussions on driving safety. Notably, our studies were conducted in generally healthy community-dwelling adults in which the frequency of spatial orientation impairment was quite low. It is likely that screening of persons diagnosed with early stage dementia, who often are most difficult to evaluate with a brief screener to estimate driving fitness, would yield a higher rate of “true positive” cases for crash risk using spatial orientation as a benchmark. 

Strengths of this analysis include use of a large population-based sample of older drivers aged 70 years and older and the validation of findings in an independent sample of high-risk drivers. Information on several important factors known to affect crash risk were included and adjusted for in the analysis. The study was strengthened by its prospective design and the use of police-accident reports to objectively define the outcome and assess at-fault status. Some limitations should be considered. Although specific cognitive functions were associated with crash risk, the exact timeframe of impairment onset is unknown. Another limitation is the low prevalence of impairment on certain cognitive domains, including orientation to place. Consequently, point estimates for the measure of association are susceptible to imprecision.

## 5. Conclusions

In summary, this study found that impairment in orientation to place measured at baseline was associated with future rates of crash and at-fault crash involvement in older drivers per mile driven. Greater attention to individual MMSE components or dimensions may prove useful to researchers and clinicians. Assessing the spatial orientation MMSE domain may assist in identifying patients at-risk for future crash involvement.

## Figures and Tables

**Table 1:** Select baseline characteristics in Licensure and KEYS participants.

	Licensure (*n* = 1995)	KEYS (*n* = 397)
Age, mean (SD)	77.2 (5.0)	73.5 (6.1)
Female, *n* (%)	872 (43.7%)	123 (31.0%)
White, *n* (%)	1634 (81.9%)	306 (77.1%)
High school or more, *n* (%)	1363 (68.4%)	285 (78.1%)
Visual acuity, logMAR (OU)		
≤0.0 (not impaired)	1832 (91.9%)	368 (92.7%)
>0.0 (impaired)	161 (8.1%)	29 (7.3%)
Annual mileage, mean (SD)	9534 (9583)	17,470 (21,987)
MMSE (# correct)		
Overall		
Impaired (<24)	46 (2.3%)	15 (3.8%)
Not impaired (≥24)	1949 (97.7%)	382 (96.2%)
Orientation to time		
Impaired (<4)	27 (1.4%)	5 (1.3%)
Not impaired (≥4)	1968 (98.7%)	392 (98.7%)
Orientation to place		
Impaired (<4)	4 (0.2%)	4 (1.0%)
Not impaired (≥4)	1991 (99.8%)	393 (99.0%)
Registration		
Impaired (<3)	17 (0.9%)	43 (10.8%)
Not impaired (3)	1978 (99.2%)	354 (89.2%)
Attention and concentration		
Impaired (<4)	96 (4.8%)	38 (9.6%)
Not impaired (≥4)	1899 (95.2%)	359 (90.4%)
Delayed recall		
Impaired (<3)	1011 (50.7%)	255 (64.2%)
Not impaired (3)	984 (49.3%)	142 (35.8%)
Language		
Impaired (<5)	5 (0.3%)	0 (0.0%)
Not impaired (≥5)	1990 (99.8%)	397 (100.0%)
Visual construction		
Impaired (0)	224 (11.2%)	63 (15.9%)
Not impaired (1)	1770 (88.8%)	334 (84.1%)

**Table 2:** Adjusted association between impairment on the MMSE domains at baseline and rate of future MVC involvement.

**MMSE (Impaired vs. Not Impaired)**	**Any MVC in Licensure**		**Any MVC in KEYS**	
	**Adjusted ^3^ RR**	**95% CI**	**Adjusted ^3^ RR**	**95% CI**
Overall	1.43	0.76–2.66	0.63	0.15–2.60
Orientation to time	1.35	0.55–3.30	---	---
Orientation to place	6.14	1.90–19.86	6.37	2.26–17.92
Registration	0.38	0.05–2.74	0.81	0.38–1.71
Attention and concentration	1.12	0.67–1.85	0.69	0.27–1.75
Recall	1.29	1.01–1.66	1.52	0.93–2.49
Language	0.93	0.13–6.73	---	---
Visual construction	1.01	0.68–1.48	1.38	0.76–2.52
	**At-fault MVC in Licensure**		**At-fault MVC in KEYS**	
	**Adjusted ^3^ RR**	**95% CI**	**Adjusted ^3^ RR**	**95% CI**
Overall	1.83	0.87–3.88	1.05	0.25–4.44
Orientation to time	1.48	0.47–4.72	---	---
Orientation to place	6.39	1.51–27.10	10.58	3.67–30.54
Registration	0.67	0.09–4.83	0.62	0.22–1.77
Attention and concentration	1.52	0.82–2.82	0.60	0.18–2.02
Recall	1.31	0.92–1.87	1.89	0.98–3.63
Language	---	---	---	---
Visual construction	1.36	0.82–2.23	1.54	0.72–3.30

^3^ Adjusted for age, gender, race, education, and visual acuity impairment status. An offset term was used to adjust for annual mileage. Abbreviations: CI, confidence interval; RR, rate ratio; MMSE, mini-mental state examination.

**Table 3:** Overall MMSE score by MMSE domain impairment status.

	Mean (SD)	Min	Max
Orientation to time			
Impaired (<4)	21.7 (4.1)	10	27
Not impaired (≥4)	28.3 (1.7)	18	30
Orientation to place			
Impaired (<4)	18.0 (5.6)	10	23
Not impaired (≥4)	28.2 (1.8)	11	30
Registration			
Impaired (<3)	24.6 (4.3)	11	28
Not impaired (3)	28.2 (1.9)	10	30
Attention and concentration			
Impaired (<4)	24.2 (3.3)	10	28
Not impaired (≥4)	28.4 (1.6)	18	30
Delayed recall			
Impaired (<3)	27.1 (2.0)	10	29
Not impaired (3)	29.3 (0.9)	23	30
Language			
Impaired (<5)	17.4 (4.0)	11	22
Not impaired (≥5)	28.2 (1.8)	10	30
Visual construction			
Impaired (0)	25.9 (2.9)	10	29
Not impaired (1)	28.5 (1.5)	18	30
